# Critical evaluation of reverse engineering tool Imagix 4D!

**DOI:** 10.1186/s40064-016-3732-x

**Published:** 2016-12-23

**Authors:** Rashmi Yadav, Ravindra Patel, Abhay Kothari

**Affiliations:** 1 UIT, RGPV, Airport Bypass Road, Gandhi Nagar, Bhopal, India; 2 AITR, Mangliya, Indore, India

**Keywords:** Reverse engineering tool, Legacy code, Visualization

## Abstract

**Introduction:**

The comprehension of legacy codes is difficult to understand. Various commercial reengineering tools are available that have unique working styles, and are equipped with their inherent capabilities and shortcomings. The focus of the available tools is in visualizing static behavior not the dynamic one. Therefore, it is difficult for people who work in software product maintenance, code understanding reengineering/reverse engineering. Consequently, the need for a comprehensive reengineering/reverse engineering tool arises. We found the usage of Imagix 4D to be good as it generates the maximum pictorial representations in the form of flow charts, flow graphs, class diagrams, metrics and, to a partial extent, dynamic visualizations.

**Case description and evolution:**

We evaluated Imagix 4D with the help of a case study involving a few samples of source code. The behavior of the tool was analyzed on multiple small codes and a large code gcc C parser. Large code evaluation was performed to uncover dead code, unstructured code, and the effect of not including required files at preprocessing level. The utility of Imagix 4D to prepare decision density and complexity metrics for a large code was found to be useful in getting to know how much reengineering is required. At the outset, Imagix 4D offered limitations in dynamic visualizations, flow chart separation (large code) and parsing loops.

**Conclusion:**

The outcome of evaluation will eventually help in upgrading Imagix 4D and posed a need of full featured tools in the area of software reengineering/reverse engineering. It will also help the research community, especially those who are interested in the realm of software reengineering tool building.

## Background

While developing any project, one uses the latest tools and techniques, but with time, they become less useful. If it is hardware, we can afford to dispose of it and buy a newer version, but in case of software, choices may not be so easily available. Thus, we need to rebuild it and in some situations enhance it, i.e., add some functions to cope with the current needs of the customer. To understand the legacy code, which was developed years ago, and rebuild it in accordance with present demands, reengineering is needed (Rogers 2010). Reengineering has two phases. The first phase is called reverse engineering and is concerned with understanding the source code (it is most valuable artifacts), deriving the design and creating the requirements. The second phase is forward engineering and is all about taking the requirements from reverse engineering and rebuilding the new software. In this paper, our focus was only on reverse engineering. Various reengineering/reverse engineering tools are available, but they are limited in their functions. All tools have merits and demerits, and their detailed evolution and comparison is available in a research paper (Yadav et al. 2014). Here, as shown in Table 1 below, we give a comparison of the tools on the basis of the input taken by the tool and the output visualized by it. Table 1 comparison of the tools on the basis of the input taken by the tool and the output visualized by it.

**Table 1:** Comparison of the existing reengineering tools

S. no	RE tools	Input/extract
1	Rigi (Muller and Kienle 2010)	Takes C, C++ code and visualizes only function and structure data type through call graph
2	Doclike viewer (Suleiman 2005)	Takes C, C++ code and extracts software artifacts and generate the document and view module by module as per user selection
3	Sniff++ (Bellay and Gall 1998)	Takes C, C++ program as an input and visualize the graph
4	Shrimp (Storey and Michaud 2001)	Takes java Program and visualizes software hierarchies, architecture with packages and class structures
5	Code crawler (Lanza 2003)	Takes C, C++, Java, Small talk and visualize source code architecture with metrics
6	Reverse Engineering tool (Bellucci et al. 2012)	Takes Web applications, transform this web application and visualizes them into model-based pattern
7	Solidsx (Auber et al. 2010)	Takes C, C++, .NET/c#, and Java code bases and visualize treemaps, table lences and hierarchical edge bundles in a single enviorment
8	Dalli (Kazman and Carriere 1999)	Takes C, C++ code as an input and extract function call, file, processes and their relationship
9	GUPRO (Ebert et al. 2002; Riediger 2000)	Take C, C++, Java, and RDBMS and visualize the graph
10	The Code Structure Visualization Tool (Saha 2013)	Takes Java code and analyze it, finally shows the hierarchical structure of the entire program
11	DEFACTO (Basten and KLINT 2008)	Takes wide programming language, C, C++, JAVA and extracts elementary facts like variable declaration, procedure or method call or control flow statements
12	COLUMB-S (Boerboom and Janssen 2006)	Takes C/C++ projects and to extracts their UML Class Model and call graph
13	Imagix 4D Bellay and Gall (1998). http://www.imagix.com	Takes C, C++ and Java software, and generate the flow chart, call graph, class diagram, task collaboration diagram and Metrics
14	Reveal Tool (Matzko et al. 2002)	Takes C++ Code and output the Class Diagram
15	PL/SQL Engineering Tool (Habringer et al. 2014)	Takes PL/SQL code, database schema with meta-data which is exported from the Oracle database and provided as comma-separated files.And Visualize the high-level representation(Graph)
16	Super Womble (Jackson and Waingold 2001)	Takes Java byte code and generate object mode
17	Pilfer (Sutton and Maletic 2005)	Takes C++ code and output the Class Diagram
18	REOffice (Yang 2003)	Integration of PowerExcelRigi take as a input program the artifacts from Rigi format program fact files, resulting from the use of Excel and reproduce Rigi Graphs in PowerPoint
19	SVGgraph editor (Kienle et al. 2002)	Takes web applications as input and visualizes the graph with the node and linked representation
20	Code to visual flowchart. http://code-visual-to-flowchart-full-version.software.informer.com	Takes C, C++, Java source code and generate the flowchart
21	WSAD (Kienle and Muller 2007)	Takes J2EE web applications and produce facts with a table based and graph based visualizer with the help of Eclipse
22	ReDA Review data Analyzer (Thongtanunam et al. 2014)	Takes web application complex code and visualizes in the form of graph
23	Solid* tools (Reniers et al. 2014)	Takes C, C++, Java, or C# code base. Visualizes the edge bundles, treemaps, table lenses, annotated text, and dense pixel

We observed that most of the tools focus on visualizing the static arrangements of the code, but do not visualize the dynamic arrangements (sequence diagram showing object interactions) of the software product. Whereas when we want to understand the code of legacy software product it is necessary to understand the dynamic arrangement of software products, the author (Prasad and Upadhyay 2015; Bellay and Gall 1998) assessed various reengineering tools, and recommended the Imagix 4D tool. We also chose Imagix Corporation’s Imagix 4D tool for its features, and as it develops maximum architecture from source code. But it does little work in dynamic architecture generation as task collaboration diagram. Therefore, there is a need to develop a full-featured reverse engineering tool, especially, to capture the dynamic arrangements of a code. We evaluated the Imagix 4D tool thoroughly and prepared a critique that will derive the requirement of full featured reengineering tool and guide the research community those who are interested in tool building. Imagix 4D (Reniers et al. 2014) is a comprehensive static source code analysis tool. It takes the code as an input and visually explores the architecture of that code. A good feature of this tool is the simultaneously display of code and visual window, and the display of relevant portions of source code through Imagix 4D’s querying capabilities. Imagix 4D is divided into different major sub areas: Source Code Analysis, Static Analysis, Metrics and Test, Delta Analysis, and Automated Documentation. Imagix 4D generates flowcharts, flow graphs, class diagrams, etc. We analyzed the tool with the help of a case study.

## Evaluatory case study of architecture developed by Imagix 4D

Different programs which were in C, C++ and Java were taken as input by Imagix 4D, flowcharts, flow graphs, class diagrams were generated. Then, we discussed the results from diverse perspectives.

### Section I

Here we took small programs such as finding out even/odd number etc., after input this programs we investigated architecture visualized by Imagix 4D.

#### Flowchart

We took some (small source codes) that were in C, C++, Java, and which took them as inputs for Imagix 4D. The flow charts were generated with the help of Imagix 4D, and analysis of visualized flowcharts’ merits and demerits were presented for the functions, which consisted of hundreds of lines of source codes. The flow charts can help to quickly grasp the internal logic of the code. Some symbols used by Imagix 4D to draw the flowchart are shown in Appendix Fig. 1. Initially, we input program (a) in Imagix 4D tool, and then we observed that it had the flexibility to generate flowcharts in three ways. First, a simple flowchart without displaying the code details is presented in Fig. 2. This flowchart gives the internal logic of the program, but not the coding details. This is suitable when the source code is too large and we are interested only in logic. In Fig. 3, we observed the flowcharts’ block contains full coding details,and no blocks of flowcharts were blank. It is very useful when we are interested in internal coding details. Although, these flowcharts are too big for large source codes. The third type of flowchart is shown in Fig. 4. It displays source code details with line number. It is very useful because line number is a good tractability feature to understand the code, especially in reverse engineering, when we do maintenance or code enhancement. Next, we took program (b) as an input and the flowchart generated is shown in Fig. 5. Following that, we took program (c), which is the same as program (b), the only difference is that we removed some variable declaration, which in turn meant that it was not complete or correct. The flowchart generated for programs (c) is shown in Fig. 6, where we observed that there was no variable declaration in the input program despite containing the assignment block, which meant it had no error detection and correction mechanism. We give as an input program (d) the generated flowchart is shown in Fig. 7 we saw that the Imagix 4D does not display the (for string m:ls) whereas the code contains the (for string m:ls), in our code also used if else conditional statements but it is not displayed in the source code means it is not work for advanced or extended loop. We gave as an input program (e) in C++ and it contains more than one function in program it required to generate the separate flowchart of individual function including main function which can be seen in Fig. 8, which display flowchart of main function, Fig. 9 which displays flowchart for calculating function, Fig. 10 which displays the function prime, Fig. 11 display the show function.

#### Class diagram

We have take some samples of codes given as an input and study the visualize architecture. Class diagram gives the static organization of software Project. The program (f) given as an input, the generated diagram is shown in Fig. 12, it is an abstract class diagram means it does not give the internal details like data type and access mode. When we do reengineering/reverse engineering we want to understand the code and this is very difficult to understand the code for person who see the limited details of class diagram as displayed. So the class diagram needs to display details about data type and access mode of member function. Imgix 4D does not draw the sequence diagram which is very useful when we do code enhancement or maintenance in reverse engineering. Sequence diagram shows the timing sequence in which object communicates with each other. Another missing feature of Imagix 4D is that it does not generate the ER diagram for understanding the legacy code ER diagram is very important. State machine diagram is necessary to capture the dynamic behavior of the software but Imagix 4D does not generate it.

### Section-II

In the previous section, the case study was with regard to small pieces of codes. Here, we took large source codes into account. gcc C parser code was taken to view the performance of Imagix 4D. As with the small codes, Imagix 4D performed equally well for the large code. It provided the flexibility to generate codes as per the user’s need in the form of flowcharts with code, without code, and with line number. The parser of Imagix 4D worked in same manner that it had done for small codes, but the visualized architectures (flowcharts) were sometimes difficult to understand due to their large size, and splitting the large output into distinct parts was also not systematic. As shown in Fig. 13, metrics visualization is another strong factor of Imagix 4D. It shows different metrics of code function such as McCabe cyclomatic complexity, McCabe decision density, Trans Fan in, etc. These are helpful for assessing design quality, performing reengineering, determining the extent to which reengineering is needed, and in software testing. It also provides the number of jump statements (goto), break or continue statements used by the program that make it more difficult to understand the unstructured code, which is an indication to reengineer the design. But some lines of code is ignored by the analyzer of Imagix 4D due to possible mistakes of not including required files at the coding level, or some syntax not being resolved by the analyzer, show. Imagix 4D does not accurately identify certain call made through function pointer in variable dependencies as shown in Fig. 14 this reports the variables and files involving code which affects values of these variables. This could support traceability. With a limitation of not reporting calls made through function pointer. It also shows the dead code which consists of root function, has no calling function, and remains unexecuted. But its detection is not fully automated, as human involvement is necessary to understand the dead code.

## Conclusion

Imagix 4D is a good tool in terms of variety of language supportability, graphical user interface, maximum diagram generation, and provide choices for visualize information by using filtering techniques. Still, there is a need to enhance the parser, especially for error detection mechanism, and to read and visualize some extended conditional statement. Class diagrams generated by Imagix 4D are not concrete in nature in terms of understanding codes. Our evaluation of Imagix 4D on the large code of gcc C parser revealed its abilities to show dead code, unstructured code, and code part written without inclusion of required file. All these support the cause of reengineering. The metrics regarding complexity, fan-in density helps to understand the design quality, and type and amount of testing need. Most reengineering/reverse engineering tools, including Imagix 4D, generate static diagram. They do not capture dynamic diagrams such as sequence and Entity relationship diagrams. They are important in code enhancement, code reuse, and reverse engineering. This study has directed research attention to build a full featured reverse engineering tool, and helps to define the requirement set of a new, full featured, comprehensive reengineering/reverse engineering tool.

## Evaluatory case presentation Appendix

### Evaluatory case study of flowchart

**Fig. 1:**
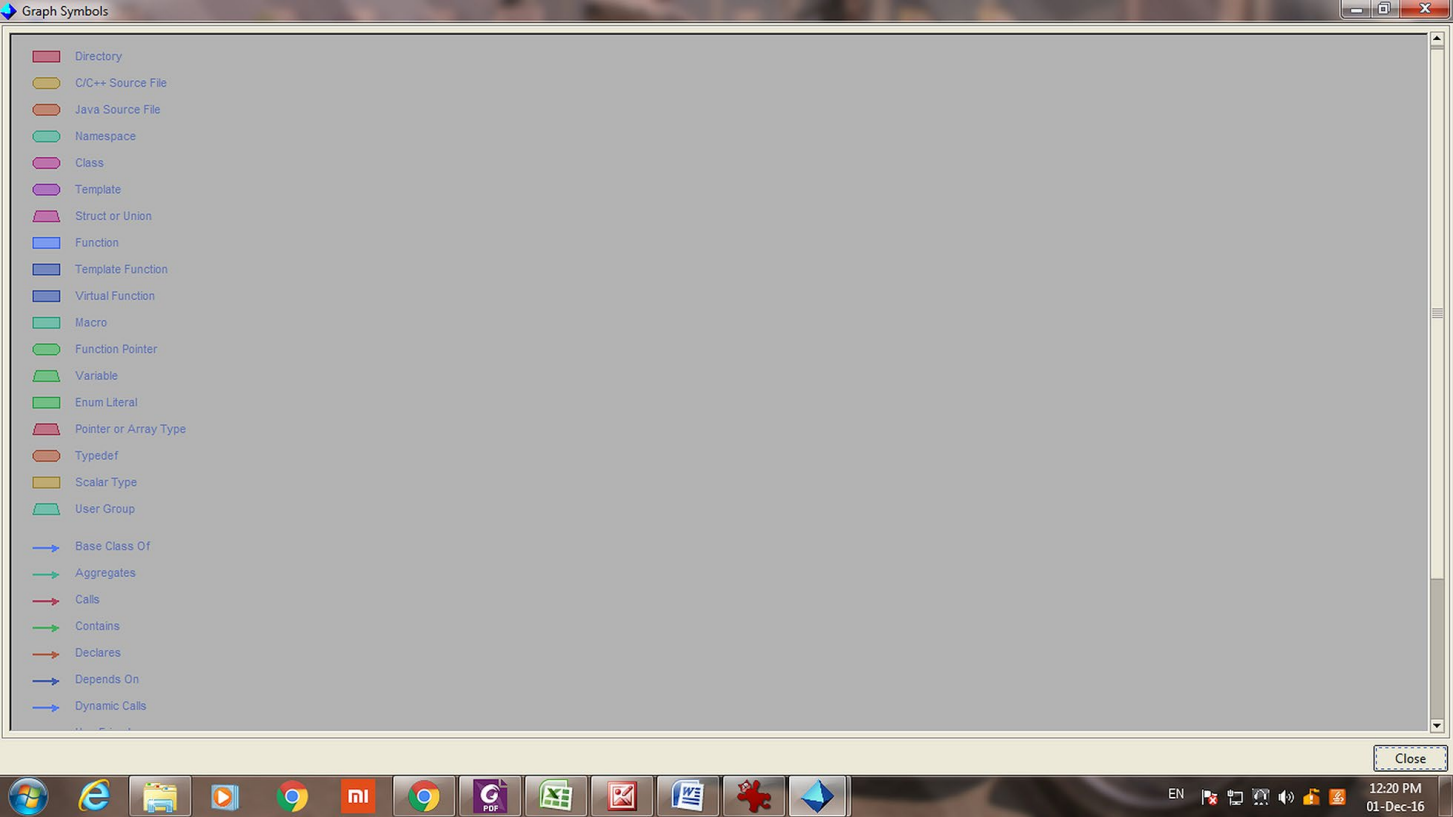
Symbol used in the flow chart by Imagix 4D

In this section, we analyzed, in detail, the different small codes taken by Imagix 4D as input, and studied the visualized flowchart. Figure 1 displays the symbols used by Imagix 4D.

- Prime number C Program This is a small code which takes a number and displays whether it is prime or not.

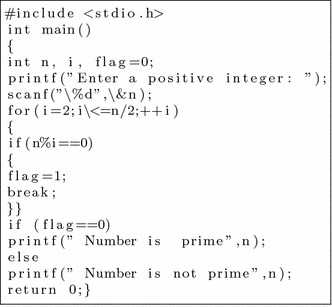


When we input above program (a) in Imagix 4D, it read that program in left to right, and top to bottom fashion and generated the flowchart. Here, we noted that it provided flexibility in generating the flow chart according to user’s choice.

### Simple flow charts without display the code details

**Fig. 2:**
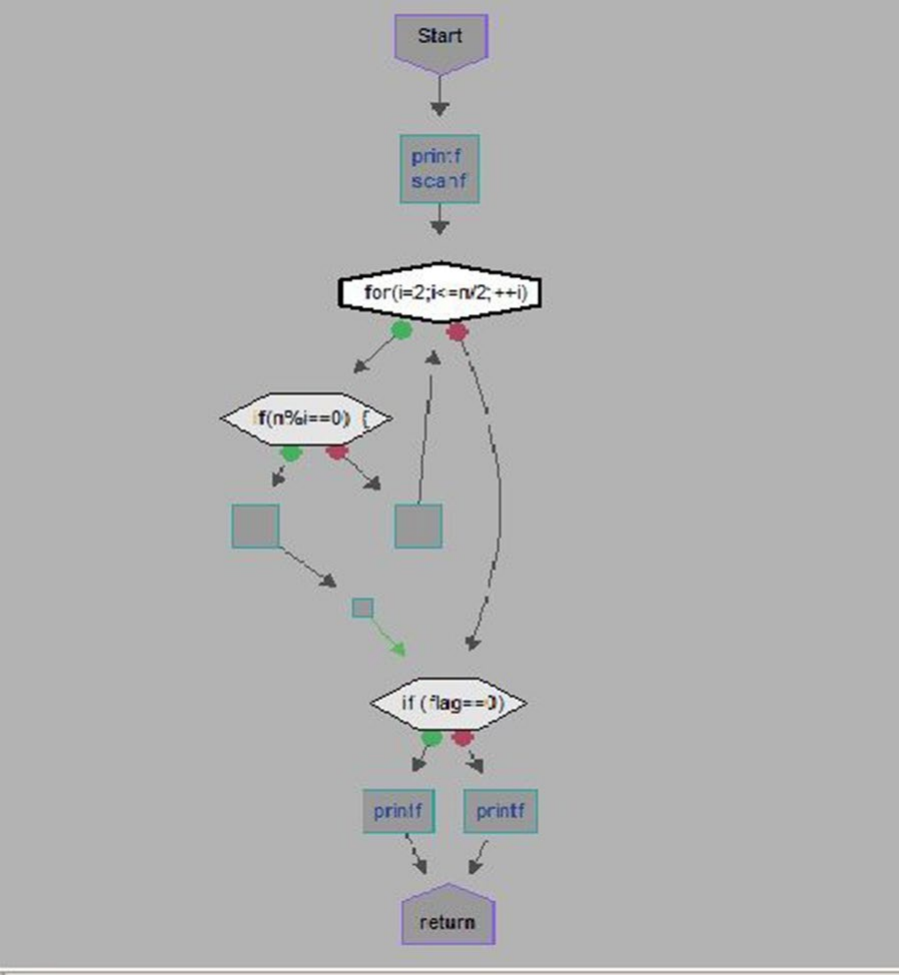
Flow chart of Prime Number Program without Source code

**Fig. 3:**
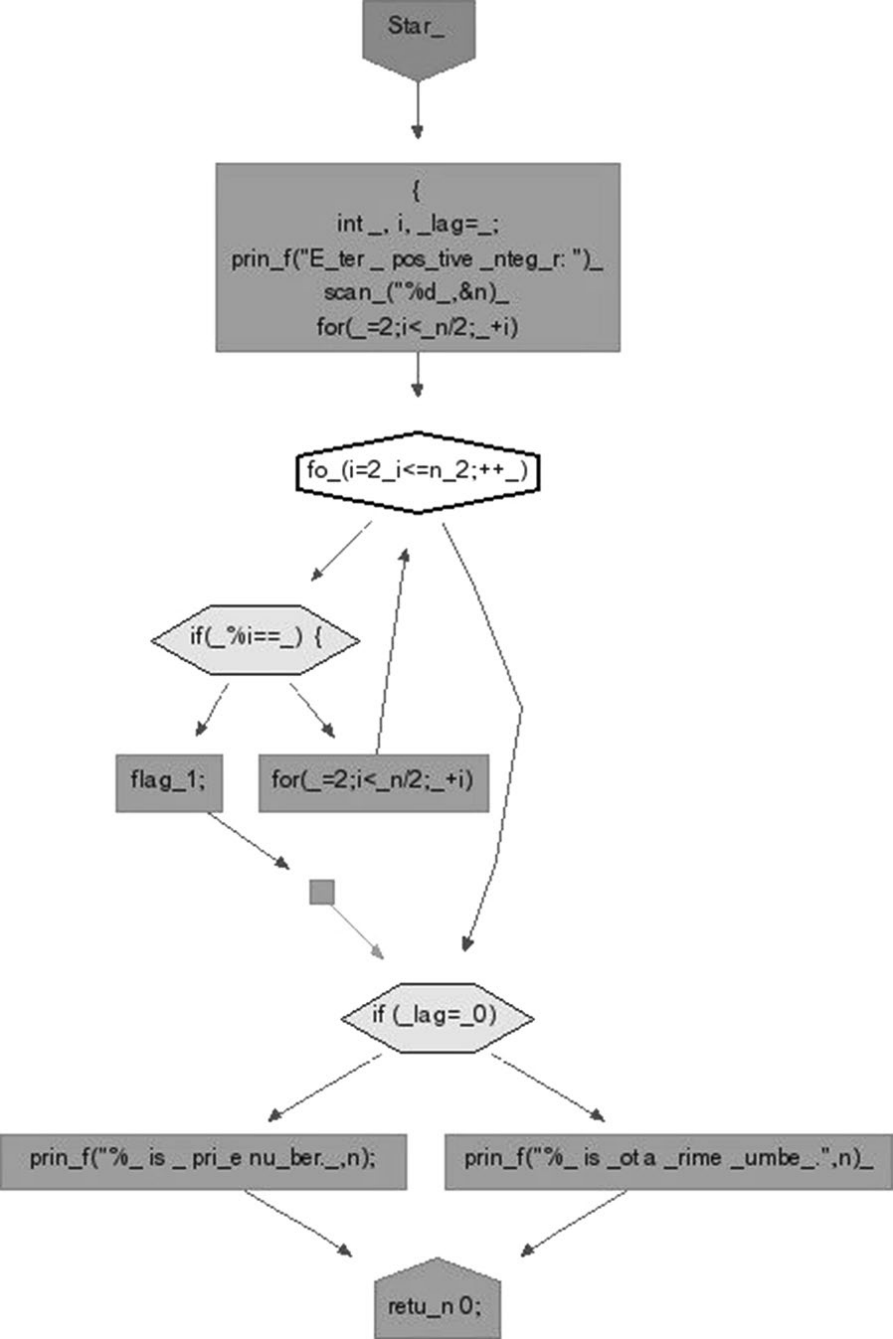
Flow chart of Prime No Program with Source code without Line Number

Figure 2 displays flowchart without source code details. Here, the flowchart gave the internal logic of the program, but not the coding details such as assignment block. It only displayed input output function. Some assignment blocks are empty. Figure 3 provides internal code details. But, when a program is very large and we want to see only the flow of programs Then the Flowcharts without source code are useful as it optimizes the flowcharts, reduces complexities, and helps in understanding the overall logic of the program.

### Flow charts displaying the code details

 In Fig. 3, the generated flow chart displays the coding parts in detail. It is useful when reverse engineers or developers want to comprehend the code details.

### Flow charts displaying code details with line numbers

**Fig. 4:**
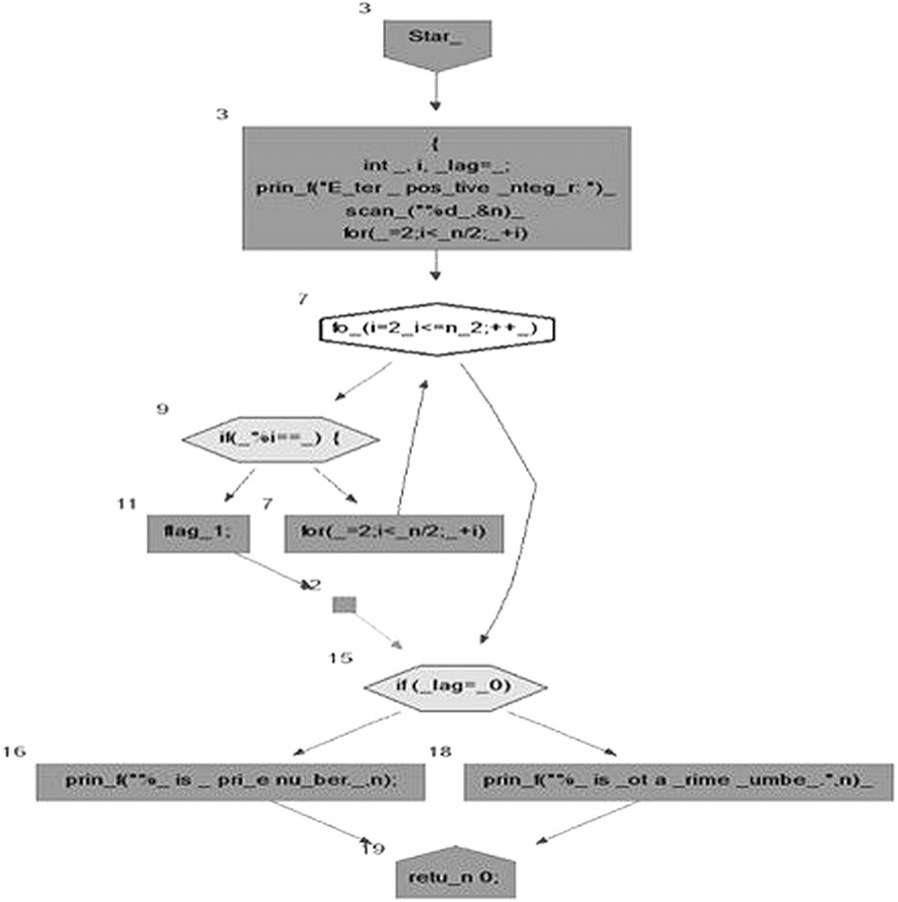
Flow chart of Prime Number Program with Source code and Line Number

In Fig. 4, the generated flow chart displays the coding parts in detail with line number. Line nuber is good traceability feature to comprehend the code details.

(b) A program to find even no in C++

This program identifies the entered number is even or not. It is implemented in C++, and checked and verified to confirm all variable declarations are taken care of.

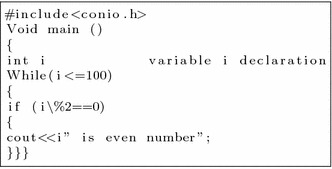



**Fig. 5:**
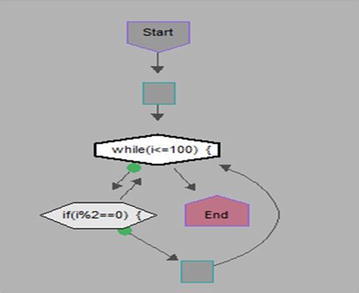
Flow chart of Program Find even No C++ with Variable Declaration

This program is inputed into Imagix 4D, and the generated diagram is studied. In Fig. 5, the generated flowchart is shown. Here, we saw the start block, then the assignment block, and finally the conditional statement. If the statement is false, it ends the flowchart. On the other hand, if it is true, it checks, again, the condition for a number to be prime. If the condition is true, the number is prime, or else the number is not prime.

(c) Program for Find even no in C++ without Variable Declaration

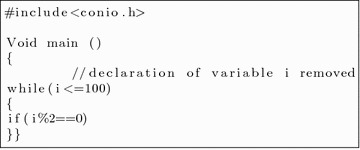

**Fig. 6:**
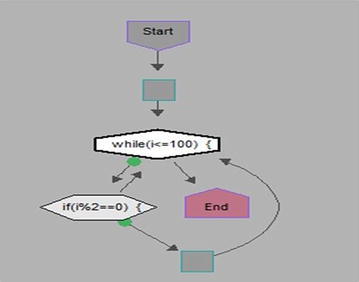
Flow chart of Program Find even No in C++ without Variable Declaration

This program is the same as previous program (b), i.e., to find out whether the entered number is even or not, The only difference is that it is not a correct or complete program, having removed the variable declaration. We gave it as an input to Imagix 4D, and studied the generated diagram. In Fig. 6, we observed that in this program, we remove the variable declaration. But it takes an assignment block for variable declaration after the start block. It has no error detection mechanism available.

(d) Program to Display Hello using pattern Matching Implemented in Java

This is a small program in java for the pattern matching. It was taken as input and study of generated diagram was studied.

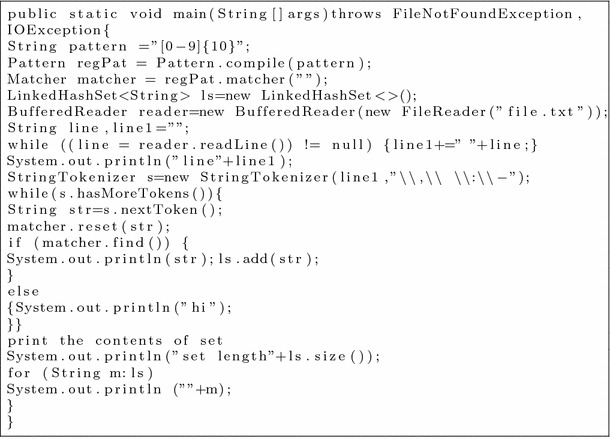



**Fig. 7:**
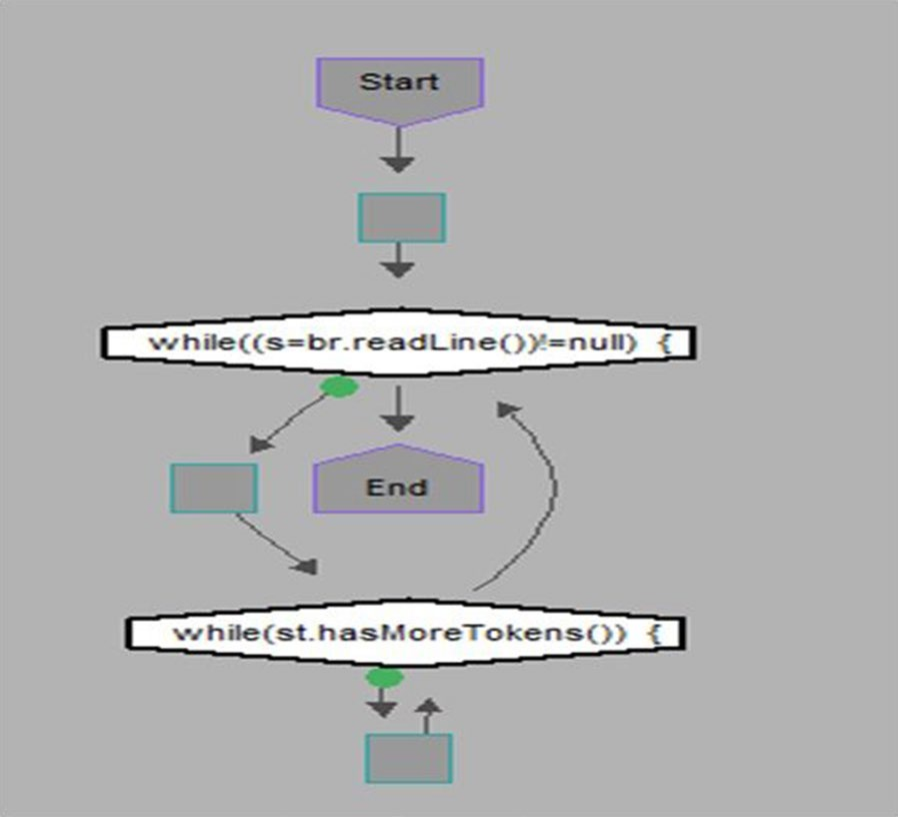
Flow chart of Program Display Hello by pattern matching in Java

In Fig. 7, we observed that Imagix 4D was not reading the (for (String m: ls)). We also used If ELSE conditional statements, but it is not displayed in the flow chart.

(e) Prime No C++

This program is taken as an input for Imagix 4D, and it is used for finding if the number is prime or not. It is the same as program (a), but it is developed in C++. The program used three user define functions Calculate (), Show () and Prime (). If user defined functions are used in the program given to Imagix 4D, it generates flowcharts separately. It reads and divides codes into different available functions, including main (), and draws flowcharts for individual functions.

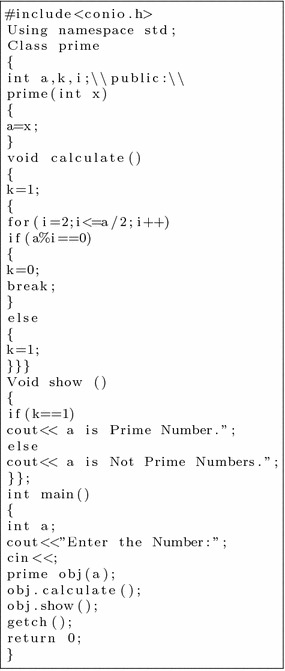



**Fig. 8:**
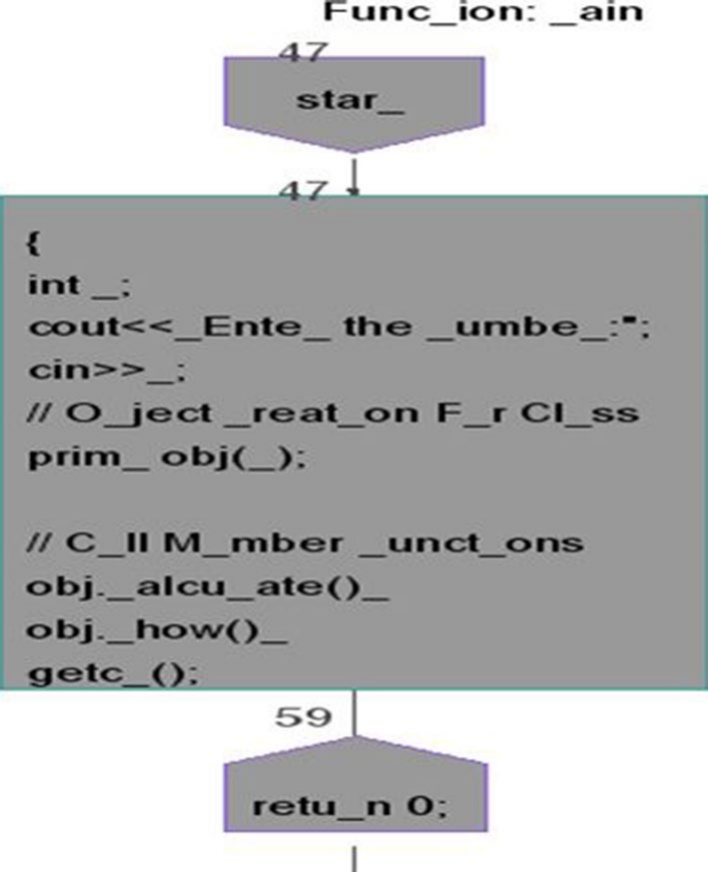
Flowchart through Imagix-4D for Prime No Program in C++ of Main Function

We found that if one or more functions are available in code, including the main function. The Imagix-4D generates separate the flow charts. Figure 8 shows the flow chart for the main function, it first put the start block and put the scope of the function. Then put all initialization and input, output statements into the initialization box, and then it uses the end symbol for return statement.

**Fig. 9:**
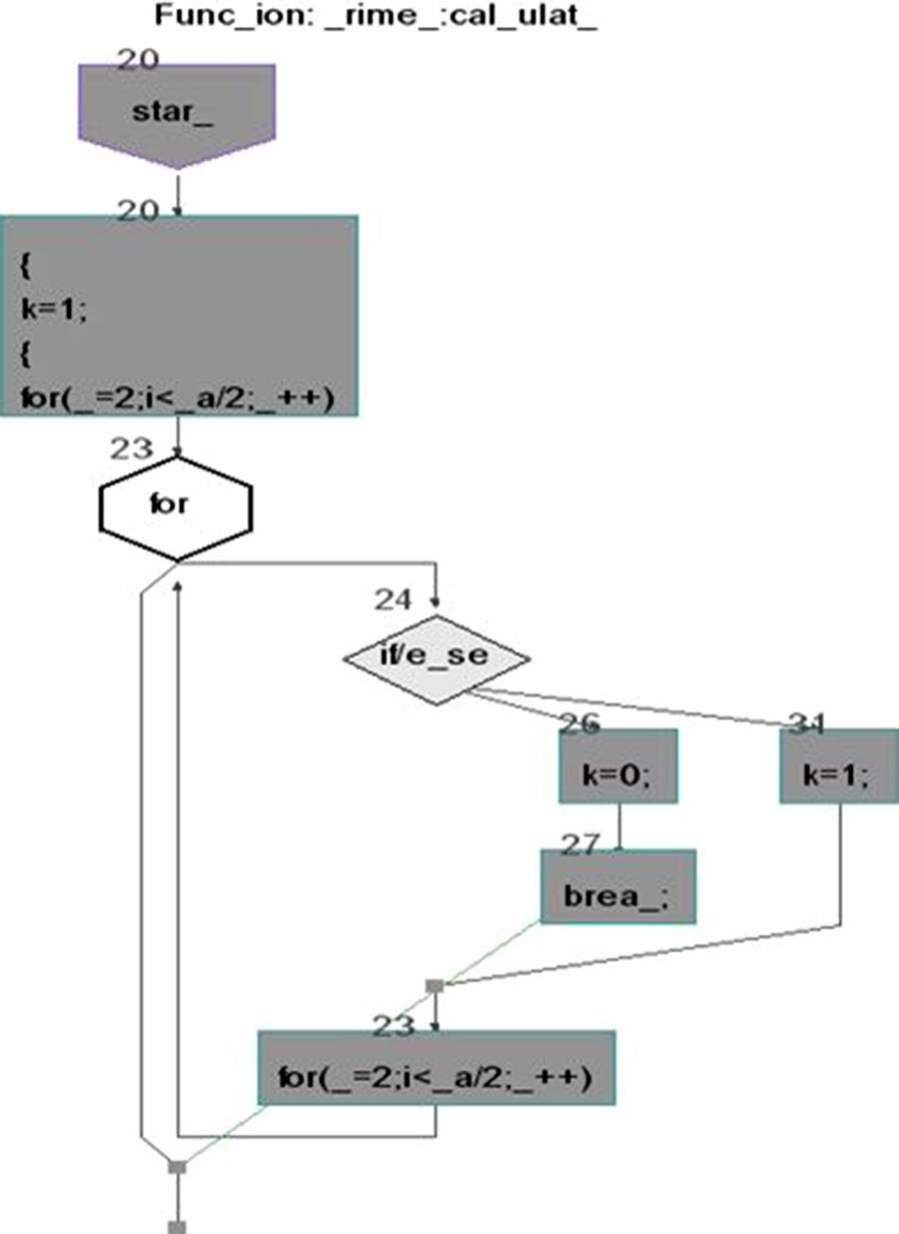
Flow chart of Prime Number Program in C++ for calculating Function

Figure 9 displays the flowchart for calculating function. It reads execution of each line and put into initialization box, then it gets the FOR loop and put into a conditional statement.One thing is observed here FOR is loop repeated two times first in initialization block and again in the conditional statement.For large and complex code this repetition create the extra overhead in code understanding.

**Fig. 10:**
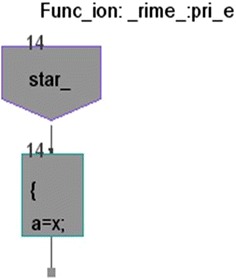
Flow chart of Prime Number Program in C++ of Prime Function

Figure 10 displays the flow chart of the prime function, tool puts start symbol,reads the body of the function that is assignment statements, put into the assignment box. Loop or conditional statements are not there so no diamond box is not used and there is no return type of the function at the end, it shows the end of scope by a small square.

**Fig. 11:**
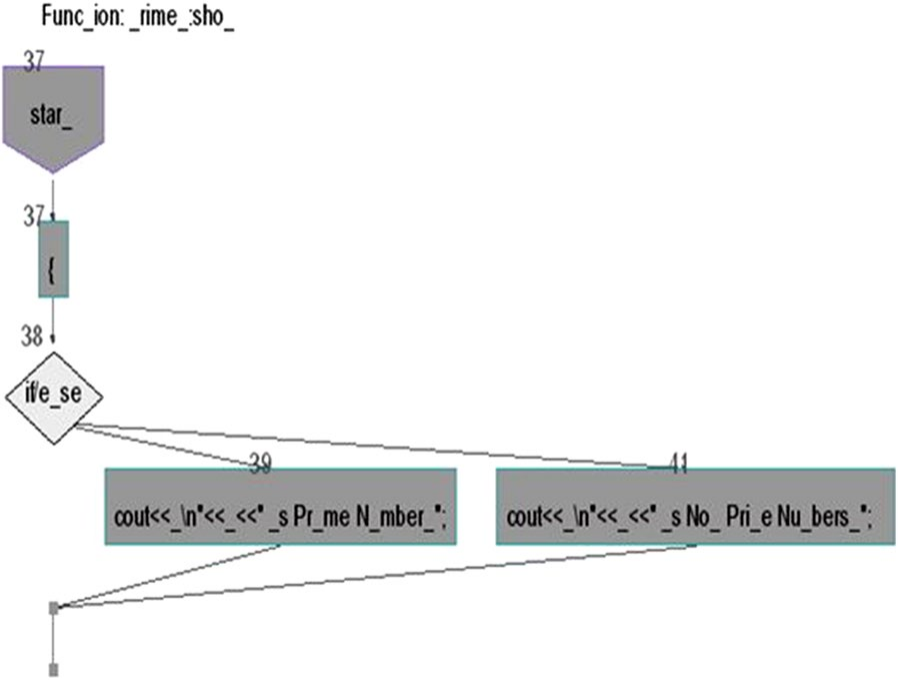
Flow chart of Prime Number Program in C++ of Show Function

Figure 11 displays flowchart of the function show in this it puts the start symbol and then there is no assignment operator and put it into the but it takes the assignment operator and put into the assignment block and displays the opening braces on that. This made the diagram bulky and put extra overhead on visualizer and person those who are interested in code understanding.

### Evaluatory case study of class diagram

In this section, we took program(f) as the sample code given as an input to Imagix-4D. The resultant diagram is further analyzed.

(f) Program in C++ to store student data

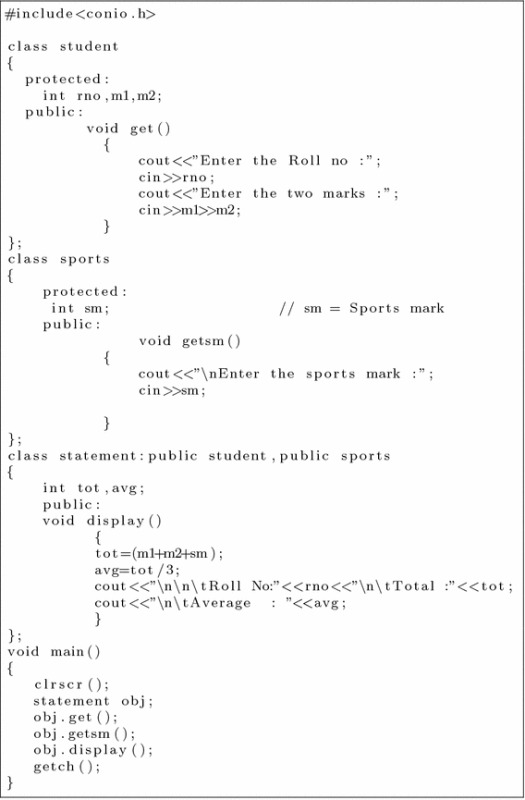

**Fig. 12:**
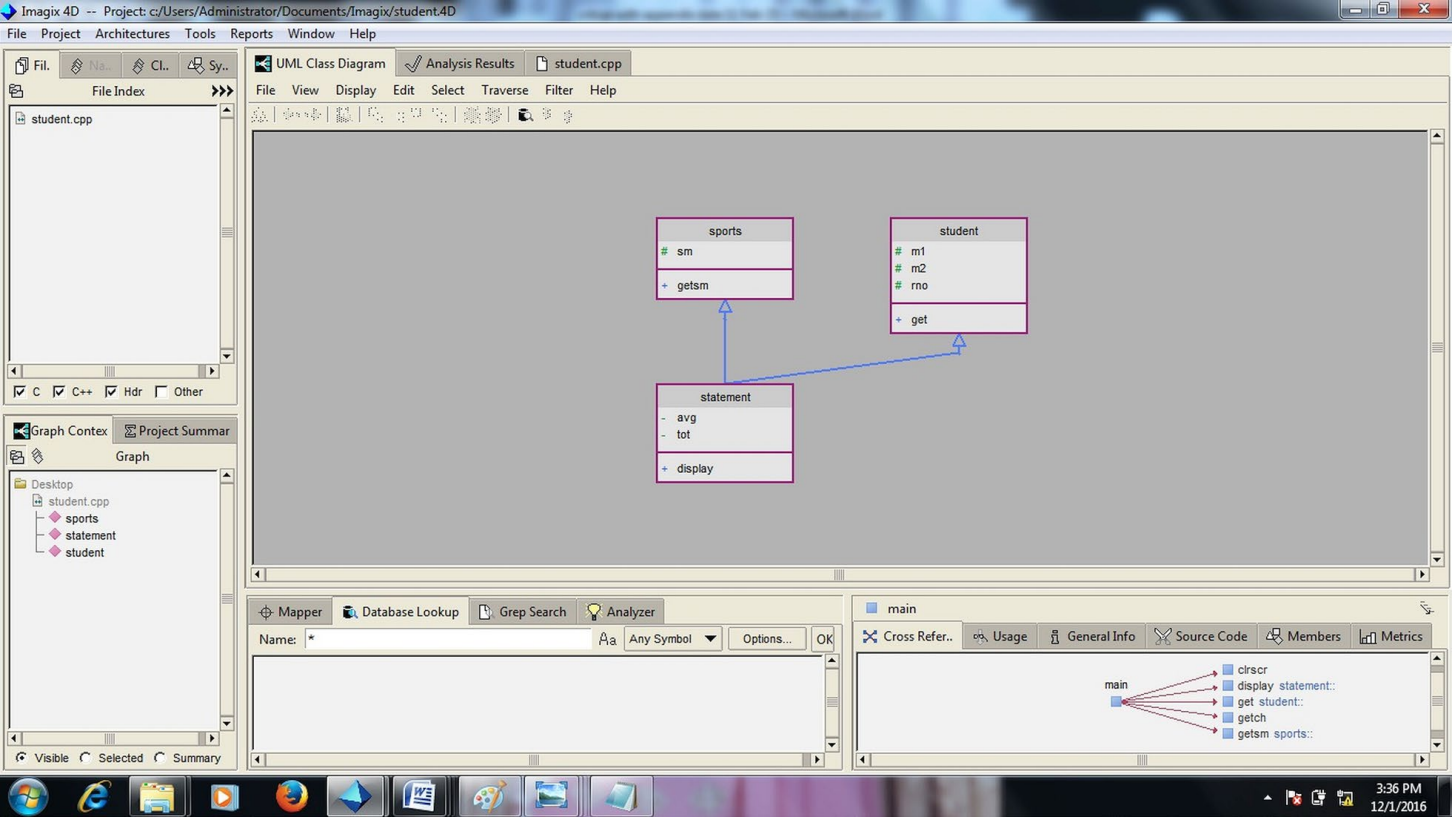
Class diagram generated by Imagix 4D

The class diagram generated by Imagix4d is shown in Fig. 12 it does not give details such as class attributes, member function, access mode and data type. These details are needed when we want to understand the code for reverse engineering of code.

### Evaluatory case study of gcc C parser program

 In this section we analyzed the gcc c parser program (large code) taken by the Imagix 4d as input and studied the visualized architecture.

**Fig. 13:**
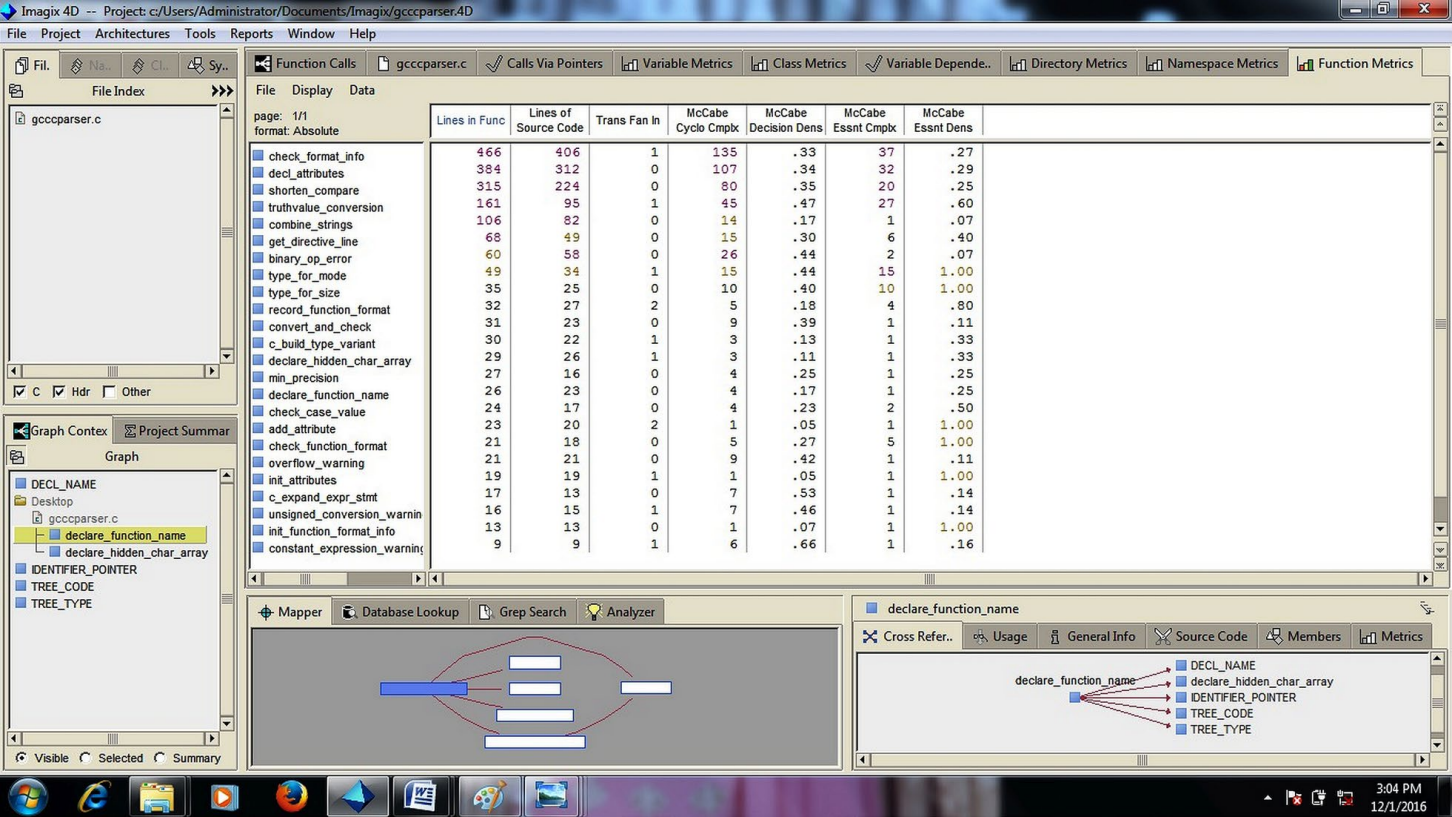
Metrics visualization diagram generated by Imagix 4D

Metric visualization is generated by the Imagix 4D as shown in Fig. 13. It is a very powerful feature of Imagix 4D. These metrics help to understand the quality of software product.

**Fig. 14:**
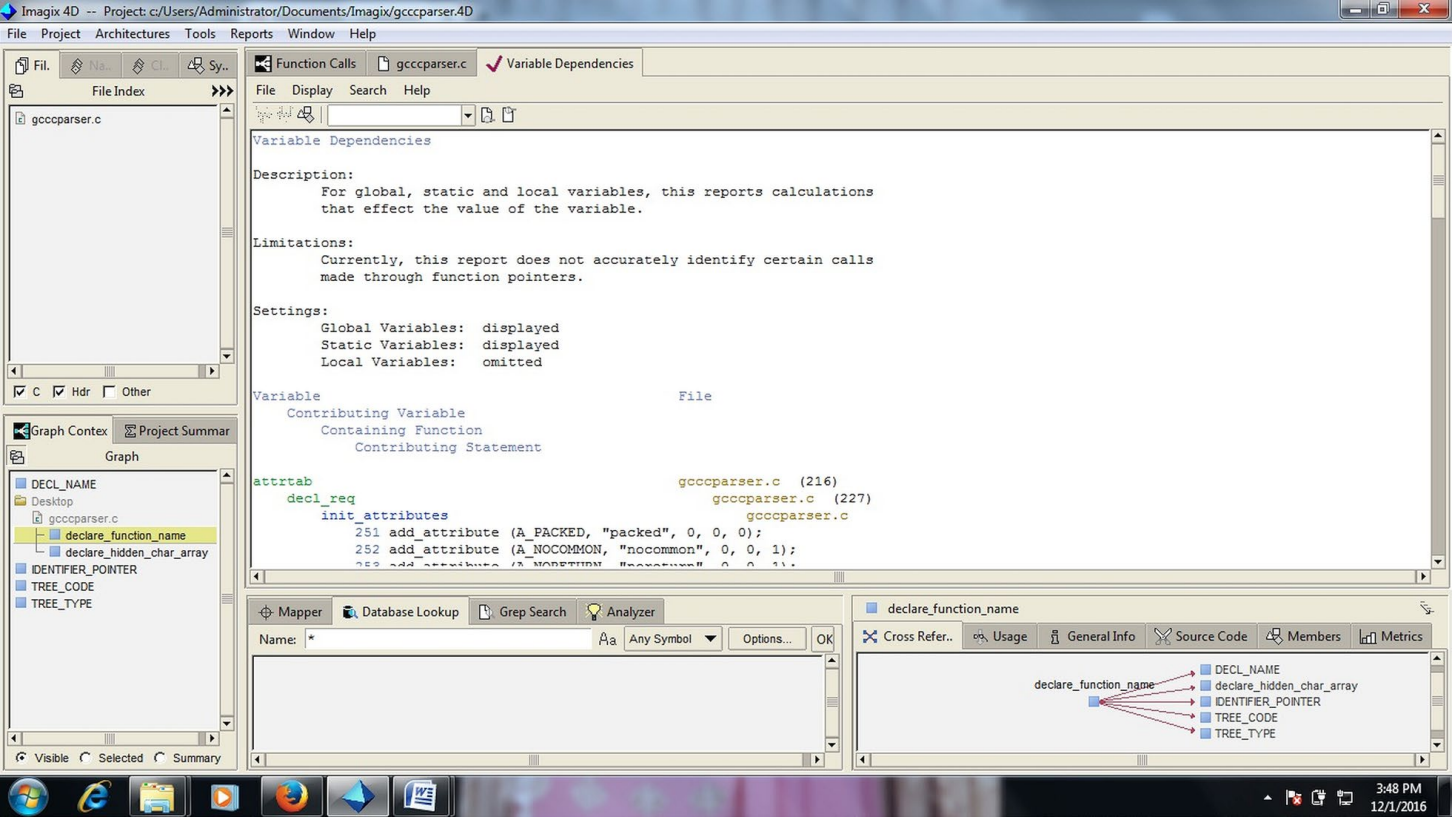
Variable dependencies generated by Imagix 4D

Variable dependencies are visualized by the Imagix 4D shown in Fig. 14. Imagix 4D is not able to accurately recognize certain call made through function pointer.
